# A novel cytotherapy device for rapid screening, enriching and combining mesenchymal stem cells into a biomaterial for promoting bone regeneration

**DOI:** 10.1038/s41598-017-15451-0

**Published:** 2017-11-13

**Authors:** Yifu Zhuang, Yaokai Gan, Dingwei Shi, Jie Zhao, Tingting Tang, Kerong Dai

**Affiliations:** Shanghai Key Laboratory of Orthopaedic Implants, Department of Orthopaedic Surgery, Shanghai Ninth People’s Hospital, Shanghai Jiao Tong University School of Medicine, Shanghai, 200011 China

## Abstract

Bone defects are a common challenge in clinic, usually warranting bone grafts. However, current strategies to obtain effective graft materials have many drawbacks. Mesenchymal stem cell (MSC)-based therapy is a promising alternative. We designed an innovative appliance named the stem cell screen–enrich–combine(-biomaterials) circulating system (SECCS). In this study, 42 patients who required bone graft underwent SECCS-based treatment. Their bone marrow samples and beta-tricalcium phosphate (β-TCP) granules were processed in the SECCS for 10–15 minutes, to produce MSC/β-TCP composites. These composites were grafted back into bone defect sites. The results showed 85.53% ± 7.95% autologous MSCs were successfully screened, enriched, and seeded on the β-TCP scaffolds synchronously. The cell viability remained unchanged after SECCS processing. Clinically, all patients obtained satisfactory bone healing. Thus, without *in vitro* culture, the SECCS can produce bioactive MSC/β-TCP composites for bone regeneration during surgery. The SECCS represents a convenient, rapid, low-cost, and safe method for bone regeneration.

## Introduction

Bone defects are a common challenge faced in the clinical setting. They are caused by various skeletal disorders, including fracture, arthrodesis, bone tumor resection, and arthroplasty revision^1,2^. These defects pose a high demand for grafting materials. Autologous graft is widely accepted as a gold standard, owing to its good osteogeneration, osteoconduction, and osteoinduction. Nevertheless, its clinical use is compromised by many limitations, such as insufficiency, chronic pain, hematoma, infection, and even fracture at donor sites^3–5^. Allogeneic graft avoids donor-site morbidity but involves risks of immune rejection and bacterial or viral contamination^6,7^. Meanwhile, artificial bone substitutes have been researched for years. However, common materials cannot replace autograft and can only be used as bone extenders^7^. Therefore, the development of new biomaterials comparable to autologous bone is urgently warranted.

Owing to their self-renewal property, multiple differentiation potential, and convenient availability, mesenchymal stem cells (MSCs) hold considerable promise in repairing bone defects. Early in 2001, 4 patients with bone defects were treated by grafting a combination of autologous MSCs and hydroxyapatite scaffold, and satisfactory long-term outcomes were obtained^6,8^. Their method represented a typical protocol of the currently used MSC-based therapy, wherein autologous MSCs are cultured *in vitro*, and the combination of expanded MSCs and scaffold is grafted into defect sites. However, expansion is time-consuming, expensive, has ethical restrictions, and poses a risk of contamination^9^. Furthermore, this procedure requires trained professionals and sophisticated facilities, as strictly recommended by good manufacturing practices^10^. Recent studies have also revealed several latent drawbacks during expansion, including decreased expression of stemness markers^11^, chromosomal aberrations^12,13^, and even malignant tumor formation^14,15^. Thus, *in vitro* expansion methods have not yet been widely accepted in clinical practice.

Based on our previous studies^1,9^, we designed an innovative approach, the stem cell screen–enrich–combine(-biomaterials) circulating system (SECCS), which takes advantage of the adherent properties of MSCs. By the circulation of bone marrow in the SECCS through porous biomaterials acting as bone marrow filters, MSCs could be rapidly screened, enriched, and combined with biomaterials. Without centrifugation or *in vitro* culture, this new non-culture technique simplifies the procedure to construct MSC/scaffold composites. Moreover, the whole process can be performed intraoperatively. In the present study, porous beta-tricalcium phosphate (β-TCP) served as scaffold. MSC/β-TCP composites produced by SECCS were grafted into human bone defects. The efficiency (to screen and enrich MSCs), safety, and clinical effect of SECCS were tested.

## Results

### Screening and enrichment rate of the SECCS

**Figure 1:**
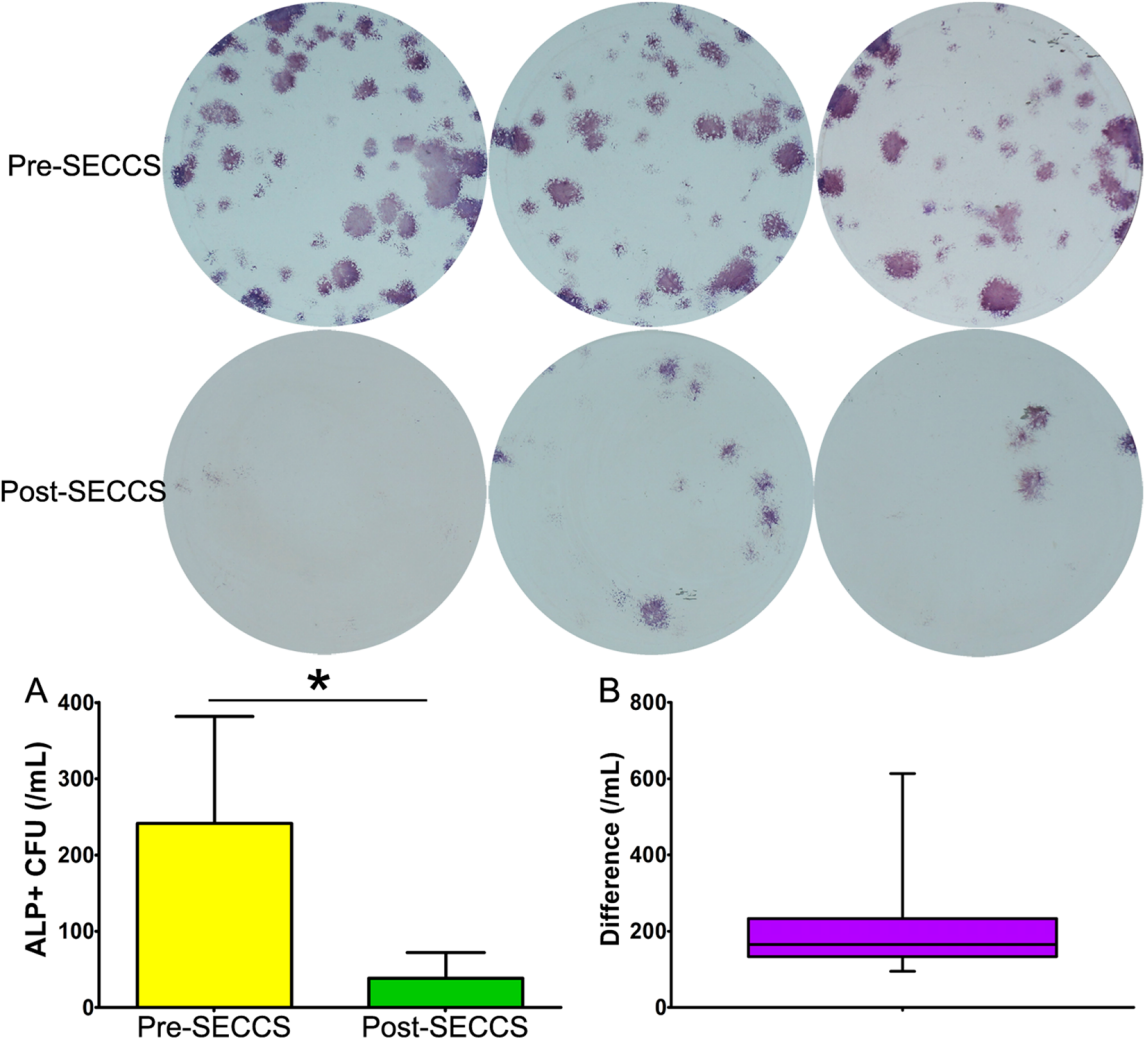
The number of colony-forming units expressing alkaline phosphatase activity (ALP + CFU) in pre- and post-SECCS bone marrow. (**A**) The number of MSCs (ALP + CFU) in pre-SECCS bone marrow was significantly higher than that in post-SECCS bone marrow (Z = −5.646, P = 0.000). (**B**) For each patient, the number of MSCs (ALP + CFU) in pre-SECCS bone marrow was higher than that in post-SECCS bone marrow. The mean decrease was 203.19 ± 117.13/mL. SECCS: screen–enrich–combine(-biomaterials) circulating system; MSCs: mesenchymal stem cells; ALP + CFU: colony-forming units expressing alkaline phosphatase activity; Difference: difference between pre- and post-SECCS ALP + CFU.

For each case, about 60 mL bone marrow was circulated in SECCS and filtered through 5 g β-TCP for 10–15 min. Before and after SECCS, ALP + CFU [colony-forming units (CFU) expressing alkaline phosphatase (ALP) activity] values of bone marrow culture were 241.57 ± 140.18/mL and 38.38 ± 33.72/mL, respectively, with a mean decrease of 203.19 ± 117.13/mL. In other words, about 12,000 MSCs were screened from bone marrow and combined with β-TCP. The normality test showed that the differences did not follow normal distribution (Z = 1.46, P = 0.028). Wilcoxon’s signed rank test showed a significant difference between pre- and post-SECCS bone marrow (Z = −5.646, P = 0.000). For all patients, ALP + CFU numbers of pre-SECCS bone marrow were much higher than that of post-SECCS bone marrow (Fig. 1). The mean screening–enrichment rate was 85.53% ± 7.95% (range, 60.25%–97.58%).

**Figure 2:**
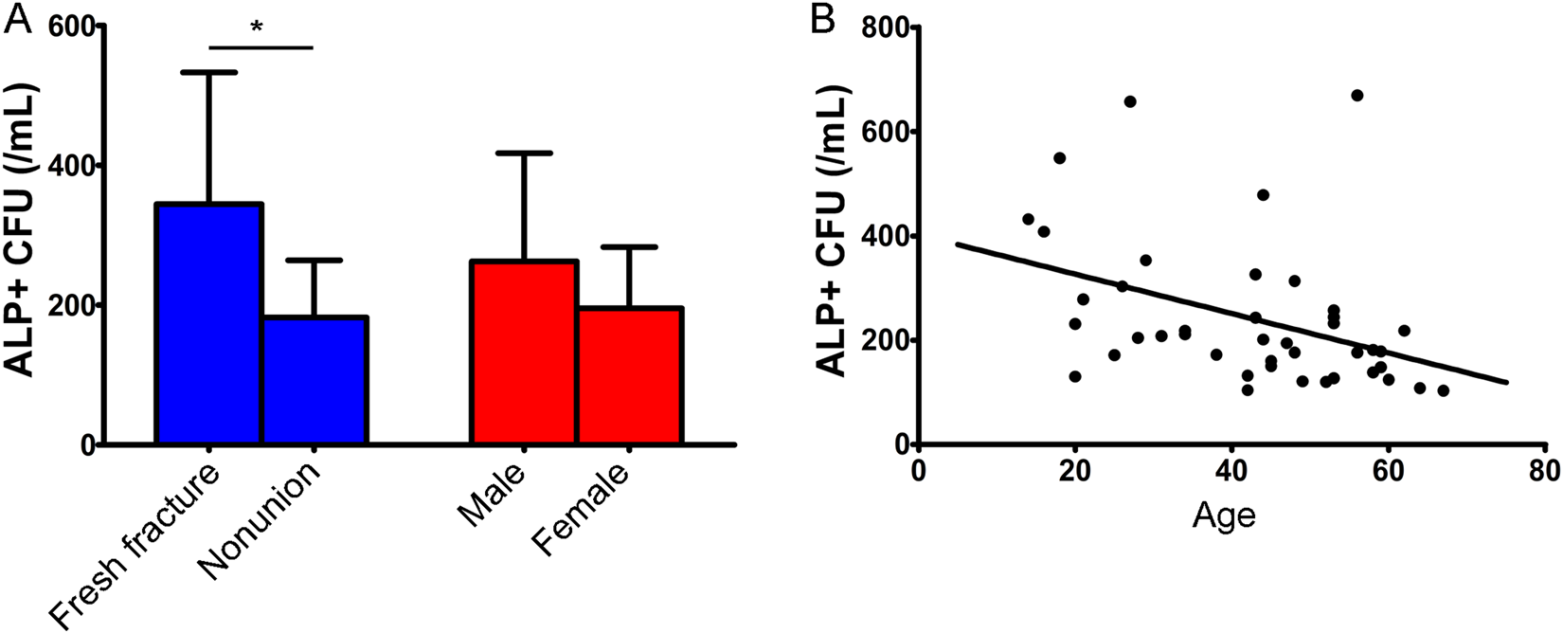
Correlation between colony-forming units expressing alkaline phosphatase activity (ALP + CFU) and individual characteristics. (**A**) The MSCs (CFUs/ALP + ) number of patients with nonunion was significantly lower than that of fresh-fracture patients (t = −0.353, P = 0.002). There was no significant difference between the MSCs (ALP + CFU) number of male and female patients (t = 1.451, P = 0.061). (**B**) The MSCs (ALP + CFU) number decreased with an increase in age (r = −0.395, R^2^ = 0.156, P = 0.01).

The MSCs (ALP + CFU) number of pre-SECCS (original) bone marrow followed normal distribution. The MSCs number in patients with nonunion (182.10 ± 82.24/mL) was significantly lower than that of fresh-fracture patients (344.64 ± 188.47/mL) (t = −0.353, P = 0.002) (Fig. 2A). Thus, fresh-fracture patients had more MSCs grafted. The MSCs number in male patients (262.31 ± 155.06/mL) was higher than that in female patients (195.31 ± 87.64), but there was no significant statistical difference (t = 1.451, P = 0.061) (Fig. 2A). A negative correlation between MSCs number of pre-SECCS bone marrow and age was noted (r = −0.395, R^2^ = 0.156, P = 0.01) (Fig. 2B).

### Nucleated cell count and cell viability

**Figure 3:**
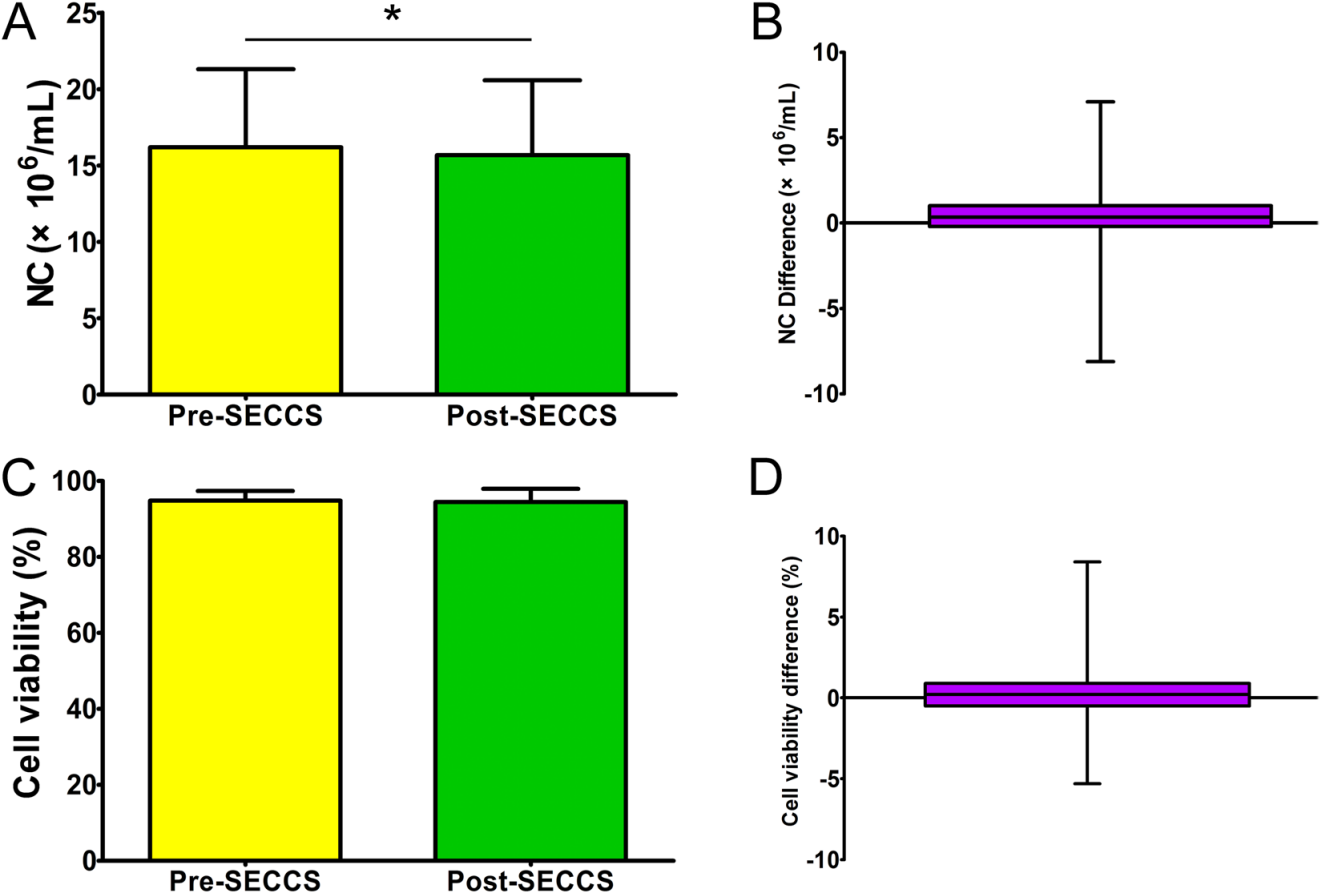
The number of nucleated cells (NC) and cell viability of pre- and post-SECCS bone marrow. (**A**,**B**) The number of NC in post-SECCS bone marrow was statistically lower than that in pre-SECCS bone marrow (Z = −2.482, P = 0.013). (**C**,**D**) There was no difference between the cell viability of pre- and post-SECCS bone marrow (t = 0.884, P = 0.382). NC (or cell viability) difference: NC number (or cell viability) difference between pre- and post-SECCS bone marrow samples.

Before SECCS processing, the nucleated cell (NC) count of bone marrow was 16.19 ± 5.12 × 10^6^/mL, and after SECCS processing, it was 15.68 ± 4.89 × 10^6^/mL. The differences did not follow normal distribution (Z = 1.44, P = 0.032). Wilcoxon’s signed rank test showed that the NC count of post-SECCS bone marrow was significantly lower than that of pre-SECCS bone marrow (Z = −2.482, P = 0.013). For most patients, NC numbers of pre-SECCS bone marrow were a little higher than that of post-SECCS bone marrow (Fig. 3A,B).

Before SECCS processing, the mean cell viability of bone marrow NC was 94.74% ± 2.54%, and after SECCS processing, it was 94.43% ± 3.48%. The differences followed normal distribution (Z = 1.27, P = 0.08), and paired t-test showed no difference between the cell viability of pre- and post-SECCS bone marrow (t = 0.884, P = 0.382) (Fig. 3C,D).

### Bacteriological examinations

All samples of pre- and post-SECCS bone marrow were monitored by bacteriological examination. Only 2 samples from 1 case were positive for bacterial infection: *Staphylococcus epidermidis* was found in both pre- and post-SECCS bone marrow of a 17-year-old boy who had osteochondroma on his right humerus. After tumor resection, the defect was grafted with MSC/β-TCP composites prepared by the SECCS. Before surgery, it was noted that he had upper respiratory tract infection in the meantime. In the first and fourth postoperative days, his body temperature was 39.2 °C and 39.4 °C, respectively. After the administration of antibiotics (cefotiam in the first 4 days and vancomycin in the following 2 days), the temperature reduced to the normal level within 1 week. C-reactive protein and procalcitonin were normal on the seventh post-operative day. No complication (swelling, increased skin temperature, aggravated pain, wound suppuration, etc.) occurred at the surgical wound site or bone marrow harvest site. Finally, he recovered completely on day 14 postoperatively and was discharged.

### Assessment of MSC/β-TCP composites

**Figure 4:**
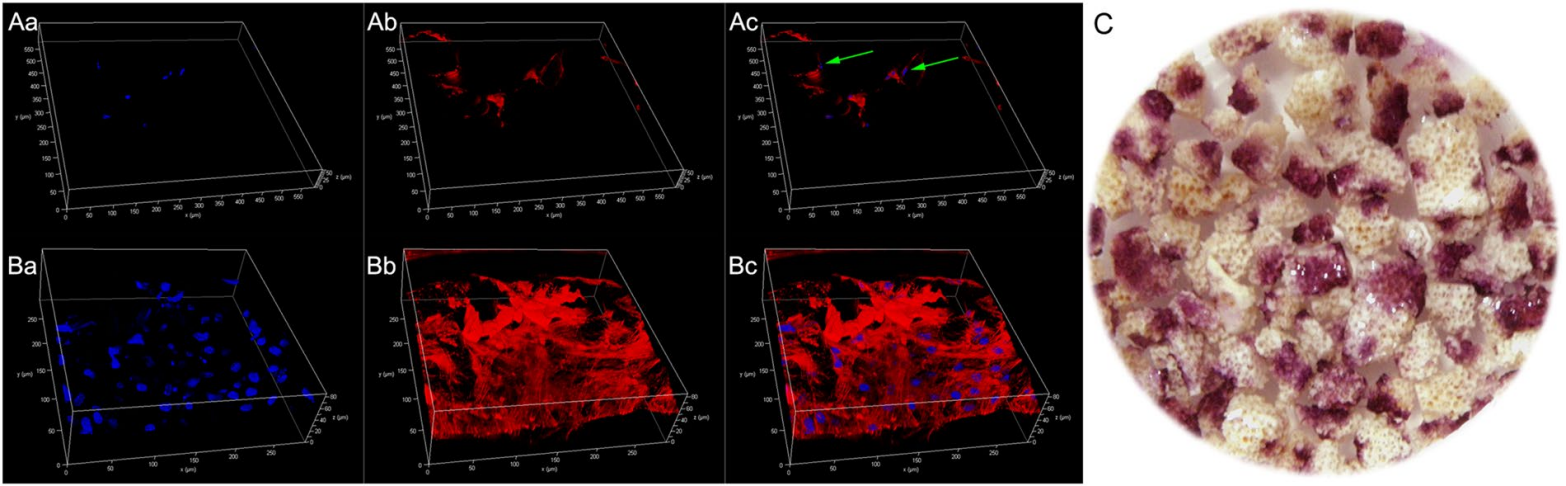
Assessment of the MSC/β-TCP composite. (**Aa**,**b**,**c**) Immediately after bone marrow circulated in the SECCS, spindle cells were found on the porous β-TCP scaffold. (**Ba**,**b**,**c**) After 10-day *in vitro* culture, many spindle cells were found on the surface and internal space of β-TCP. (**C**) After 10-day *in vitro* culture, ALP staining of MSC/β-TCP composites showed red colonies, indicating ALP activity.

After bone marrow was cycled in the SECCS, confocal microscopy showed some spindle cells adhering on the surface of and inside the porous β-TCP scaffold. After 10-day culture, a large number of spindle cells were observed on the β-TCP particles. This demonstrated that MSCs proliferated well on the β-TCP. This was also confirmed by ALP staining of the MSC/β-TCP composite, with many ALP-positive colonies (Fig. 4).

### Flow cytometry

**Figure 5:**
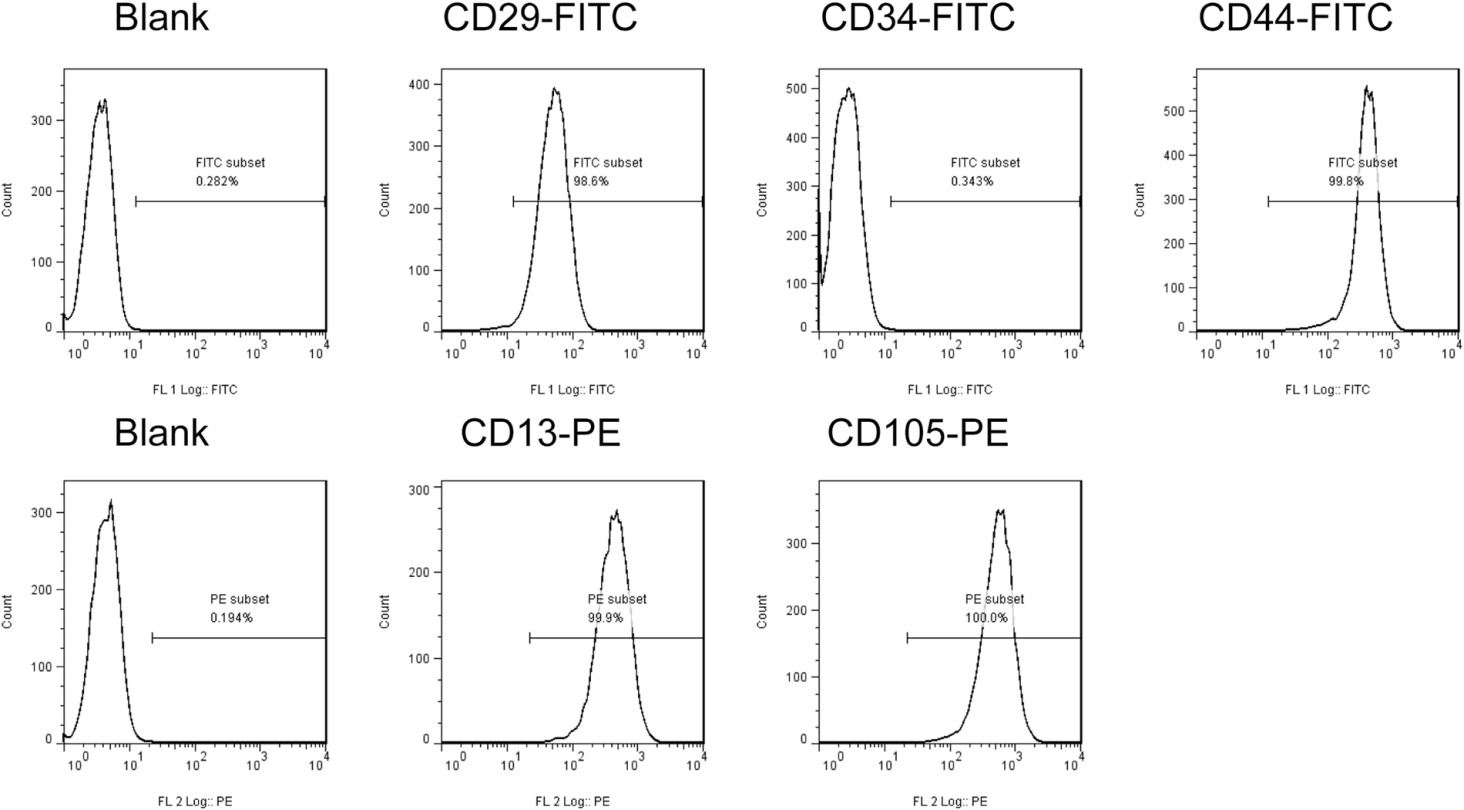
Flow cytometry. Mesenchymal stem cell markers were determined by flow cytometry.

MSC/β- TCP composites were culture *in vitro* for 21 days. And cells adhered on β-TCP were detected by flow cytometry. The results showed that these adhesive cells highly expressed CD29, CD44, CD13, and CD105; while scarcely expressed CD34 (Fig. 5). Therefore, these cells were believed to originated from MSCs which adhered to β-TCP during SECCS processing.

### Clinical outcomesg

The quantity of bone marrow harvested was about 81.88 ± 7.65 mL, which took 13.2 ± 4.3 min before the routine surgery. During the bone marrow harvest, no obvious hemodynamic change or complications, such as severe bleeding, visceral injury, or needle breakage, occurred. Postoperatively, no complication occurred at bone marrow harvest site. No wound infection has happened. Wound exudation occurred in 2 patients. Both of them recovered after dressing change.

**Figure 6:**
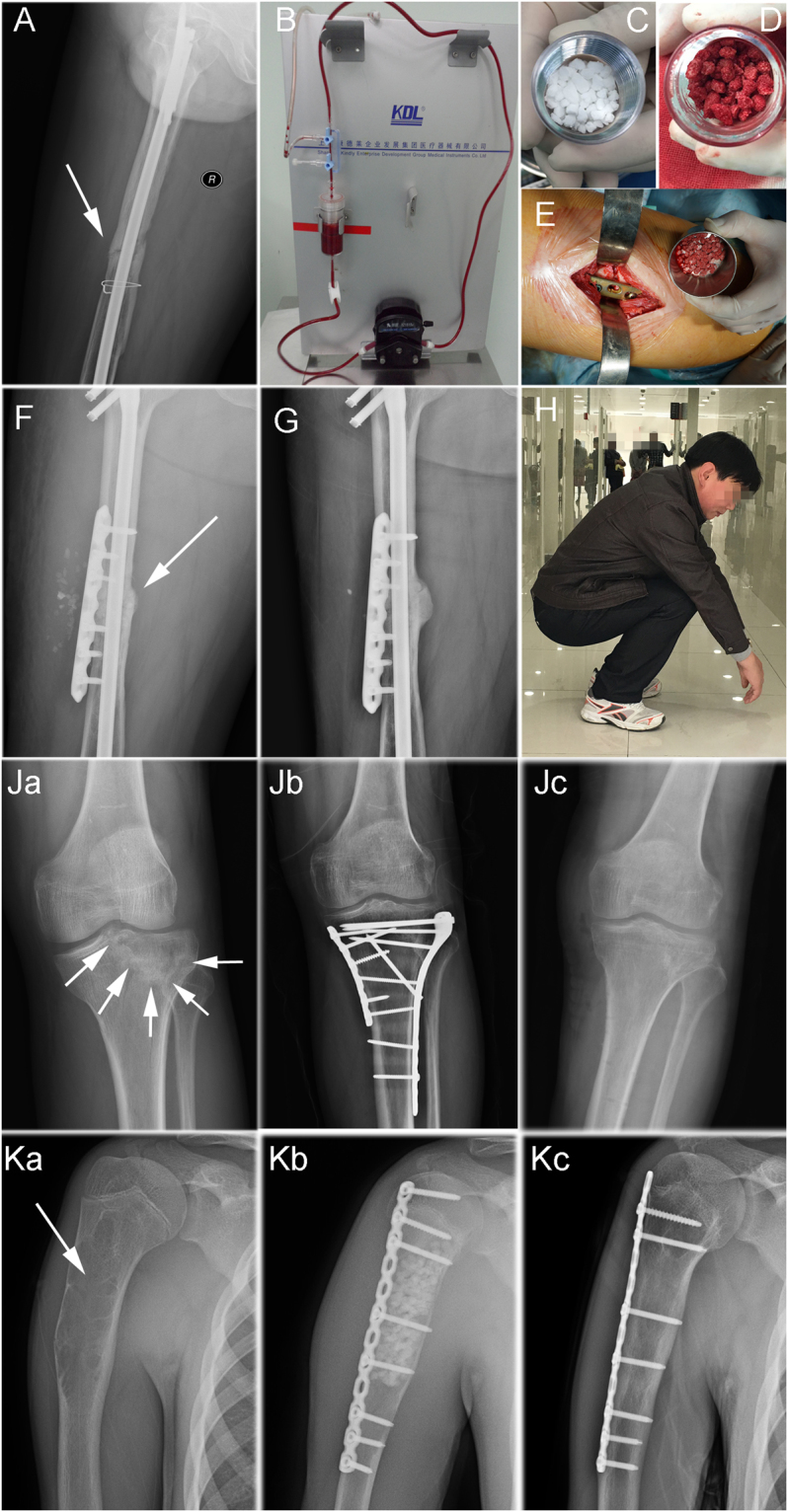
Clinical cases. (**A**) A 38-year-old man had a right femoral shaft fracture, which was treated close reduction and intramedullary nail fixation. For the next 5 years, the fracture did not heal. The fracture line was still obvious in radiograph. The patient needed a pair of crutches to facilitate walking and suffered from pain during walking. (**B–D**) β-TCP granules (white) served as filtration materials in the inner box of SECCS. Bone marrow circulated in the sealed circulatory pipe of the SECCS for 10 min. Then MSC/β-TCP composites (red granules) had been prepared. (**E**) After exposure of nonunion, the instable fracture was stabilized with a plate. The MSC/β-TCP composites, rather than autologous bone, were implanted into and around interfragmental gap. (**F**) Immediate post-operative image: the unstable fracture was fixed by a plate and grafted with MSC/β-TCP composite only. (**G**) Six months postoperatively, the nonunion completely healed, and most β-TCP particles had degraded. (**H**) The function of the injured limb was fully restored. Patient went back to work. (**Ja**) A 48-year-old female suffered a fresh fracture of right tibial plateau. (**Jb**) She underwent open reduction and internal fixation. Intraoperatively, a critical-size cavity formed after fracture reduction (white arrows). The defect was grafted with MSC/β-TCP composite. (**Jc**) After 3 months, the fracture healed. (**Ka**) A 17-year-old male was diagnosed as right humerus fibroma. (**Kb**) He was treated with tumor resection, internal fixation, and MSC/β-TCP composite graft. (**Kc**) Six months postoperatively, β-TCP granules degraded completely and graft site was restored by newly forming bone.

All patients completed follow-up with a mean duration of 17.3 months (range: 9–24 months). All patients had healed with a mean time of 5.04 ± 2.11 months (range: 2.5–9 months). (Fig. 6) In detail, the mean healing time for fresh fracture, nonunion, and other patients were 3.12, 6.29, and 4.72 months, respectively.

## Discussion

MSCs account for a minor proportion of bone marrow NCs^16^. As bone marrow filtered through porous β-TCP in the SECCS, most NCs could pass through the porous biomaterial smoothly. However, BMSCs number had decreased sharply by 85.53%. Confocal microscopy imaging showed that some spindle cells adhered to β-TCP (Fig. 4A). These cells possess proliferative ability under *in vitro* culture (Fig. 4B). Furthermore, after short-term osteogenic induction culture, ALP staining of the MSC/β-TCP composite illustrated the osteogenic differentiation potential of these adherent cells (Fig. 4C). And flow cytometry demonstrated that these cells highly expressed MSCs markers. In other words, MSCs were screened out of bone marrow and adhered on the β-TCP by rapid filtration. This was an intriguing finding. Previous studies have proved that human MSCs present dynamic adhesion on vessel endothelium during flow in a vessel^17^. Similarly, BMSCs can rapidly adhere to the surface and inner space of porous β-TCP during *in vitro* flow. The underlying mechanism needs further study. However, by taking advantage of this rapid adhesion property, the SECCS was designed to efficiently construct a bioactive MSC/β-TCP composite in the clinic.

Generally, enriched BMSCs are harvested by collecting the mononuclear cell layer, which contains most BMSCs after bone marrow centrifugation^18^. By traditional centrifugation, about 78% BMSCs are collected^9^. The SECCS combines screening with enrichment without centrifugation. As bone marrow filters through β-TCP, most of the BMSCs gather on local materials. In this manner, over 85% BMSCs were collected.

Thus far, no perfect method is available to screen BMSCs in the clinic. Current strategies include the whole-marrow adherent method^19^, density-gradient centrifugation^16,19^, flow cytometry^20^, and immunoselection^21^. The whole-marrow adherent method is simple and efficient. However, *in vitro* culture has ethical restrictions because of mixture with heterologous serum. Density-gradient media, such as Ficoll and Percoll, are commonly used in the second method. In fact, Ficoll and Percoll are cytotoxic, decreasing the proliferative activity of BMSCs^16,19^. Although the latter 2 methods yield highly purified BMSCs, they are expensive and technically complex. Moreover, flow cytometry damages the viability and functions of BMSCs^22^. In addition, the Food and Drug Administration has published guidelines for clinical stem cell treatment, which stipulate minimal manipulation within a limited time^23^. Thus, *in vitro* expansion and adding harmful reagents go against these rules. In comparison, the SECCS presents good feasibility for clinical practice. First, the SECCS is an efficient system integrating BMSC screening, BMSC enrichment, and BMSC/β-TCP composite formation, all within 10–15 min. Second, the system is convenient and easy to use in practice. All procedures can be completed during anesthesia in the same operation room. Except for bone marrow harvest, the following procedures and conventional surgery can be conducted simultaneously. Third, the SECCS is a BMSC-friendly device, dispensing with *in vitro* expansion and extrinsic reagents. Furthermore, the low-frequency peristaltic pump propels bone marrow to circulate at a low velocity, reducing mechanical damage to cells.

The SECCS confers good clinical safety. The contaminative risk during transportation should be avoided. All procedures of the SECCS-based cytotherapy are performed in laminar flow operating rooms, and the pipe of the SECCS is disposable. It provides a sealed circulatory loop for the construction of BMSC/β-TCP composites. In our series, 97.6% (41/42) samples were negative in bacterial examination. Only 1 patient had bacterial-positive results in the pre- and post-SECCS samples. This finding can be ascribed to 3 possibilities. First, the bacteria might come from hematogenous spread of his previous upper respiratory infection. Second, the bone marrow harvest process might not have been completely aseptic. The third possibility, albeit with a very low probability, was that the contamination occurred during the manipulation of the bone-marrow bacterial examination. However, because of bacterial-positive results in both pre-and post-SECCS samples, it is unlikely that contamination occurred during the bone marrow process in the SECCS; if so, the contamination would only occur in the post-SECCS bone marrow sample. Although the patient was eventually free of the infection, sterile manipulation of the bone marrow harvest and the SECCS process are paramount.

β-TCP is a popular bone graft material, which possesses excellent properties of osteoconductivity, biocompatibility, and degradability^9,24^. In this study, these small porous granules were chosen as the filtration material in the SECCS because they have a larger surface area than blocks. In the SECCS, β-TCP acts as an obstacle to intercept MSCs when bone marrow filters through these biomaterials. Hence, the greater surface area contacting the bone marrow increases the opportunity for BMSC adhesion. Moreover, the β-TCP granules are highly porous, and their internal pores are highly interconnected. This leads to a further increase in the area of contact. As bone marrow cycles in the SECCS and filters through β-TCP, most BMSCs adhere to the surface and inner walls of β-TCP. In the fracture-healing phase, β-TCP serves as a scaffold for cell proliferation, and its degradation products contribute to local mineralization^25^. A series of radiographies showed new bone formation accompanying the degradation of β-TCP.

Bone defect, especially nonunion, has resulted in a heavy healthcare burden^26^. The standardized treatment for this clinical dilemma is fracture fixation combined with autologous bone graft^27^. However, in comparison with bone marrow harvest, autologous bone harvest is more aggressive and more susceptible to complications^5^. Hence, the development of MSC-based therapy would relieve suffering of patients. In this study, there was no complaint about discomfort on bone marrow harvest site. Nowadays, *in vitro* expansion is a common strategy to obtain a large number of MSCs^28,29^. But among other drawbacks, it is time-consuming course which generally costs 8 to 28 days before MSC graft^12,30–32^. SECCS provides a simple but effective method. Because *in vitro* expansion is abandoned, from bone marrow harvest to MSCs graft, all manipulations are completed during one period of anaesthesia. Even, SECCS can be used in emergency surgery. Yet its clinical effect did not compromise by the numerical shortage. In the present work, patients were only grafted with MSC/β-TCP composites rather than autologous bone. The clinical results showed that these bioactive composites produced by SECCS are capable of osteogenesis-promoting effect in treating various diseases. Although fresh fracture patients had more MSCs grafted than nonunion patients, all nonunion patients healed within 9 months. Actually, diverse non-culture methods have been successfully carried out in many studies, such as enrichment technique^1,9^ and bone marrow concentration injection^18^. In this study, fresh primary MSCs were grafted into the defect site, resulting in good clinical outcomes. Therefore, *in vitro* expansion may be dispensable in MSC-based cytotherapy.

We also found that the number of BMSCs varied greatly between different individuals, i.e., the BMSC pool in bone marrow was affected by multiple factors. As expected, MSC number tends to decrease with age (Fig. 3B). Besides, the proliferative capacity and osteoblast differentiation potential of BMSCs also reduce during aging^33^. Therefore, the curative effect of autologous MSC therapy may be compromised in elderly patients. Notably, patients with fresh fractures had more MSCs in the bone marrow reservoir (Fig. 3A). This can be explained by MSC mobilization due to fresh fracture^34,35^. MSC mobilization promotes bone marrow cell proliferation, increasing the number of BMSC in the bone marrow. Meanwhile, MSC mobilization also enhances MSC homing (MSC are recruited from bone marrow niche and migrate to injured sites), increasing the number of peripheral blood-derived MSC^34^. Therefore, for patients with fresh fracture or other condition which could significantly mobilize MSC, peripheral blood may be an alternative to bone marrow to screen and enrich MSC using SECCS. In addition, the mean MSC number was higher in men than in women, but this difference was not significant. In fact, in the sampled individuals, more men than women happened to have undergone acute trauma. Seven of 11 fresh fracture patients were male. Moreover, Mathieu and colleagues reported that there was no difference in MSC number between healthy men and women^36^. Therefore, MSC number might be independent of sex.

## Conclusion

In summary, the SECCS is an innovative cytotherapy device, which can rapidly screen and enrich bone marrow MSCs and produce a bioactive MSC/β-TCP composite peri-operatively without *in vitro* culture. The process of the SECCS does not influence cell viability. When β-TCP served as the filtration material, bone marrow MSCs rapidly adhered to the surface and inside the porous biomaterials. A typical case showed the MSC/β-TCP composites to be capable of enhancing bone regeneration in delayed union. Thus, SECCS might represent a convenient, rapid, low-cost, and safe method for bone regeneration. However, the MSC number in the original bone marrow is influenced by disease and age. Although the all patients studied herein healed completely, large-sample randomized controlled trials using this novel cytotherapy technique are necessary to further assess its clinical effect.

## Materials and Methods

### Patients

SECCS is classified as class III medical appliances by China Food and Drug Administration. This is a single-center, non-controlled, and prospective study. The primary objective was to assess the efficiency of SECCS to screen and enrich MSC and its clinical effect. Secondary objective was to evaluate the safety and feasibility of SECCS. The Shanghai Ninth People’s Hospital Ethics Committee provided ethics approval. Both clinical treatments and laboratory experiments were carried out in accordance with relevant guidelines and regulations. Informed consent was obtained from all participants and/or their legal guardians. The study has registered on the Chinese Clinical Trial Registry, one member of WHO International Clinical Trial Registry Platform. Registration number is ChiCTR-ONC-17011448 (date: 20^th^ May 2017).

The inclusion criteria were (1) age between 15 and 65 years, with no limitation of sex; (2) bone defect patients needing surgical intervention and bone graft; and (3) patients who provided informed consent. The exclusion criteria were (1) malignant tumor, (2) severe infection, (3) hematopoietic system disorders, (4) age over 65 years or below 15 years, (5) infective surgical area, and (6) pregnant women.

From October 2013 to September 2015, 42 patients who needed bone graft surgery were enrolled in this study. The patients included 29 men and 13 women (mean age, 42.5 ± 14.62 years; range, 15–65 years). The baseline diseases of the patients are provided in Table 1. For each patient, about 80 mL bone marrow was harvested. As bone marrow circulated in SECCS, 60 mL bone marrow filtered through β-TCP granules for 10–15 min. Laboratory experiments were performed to assess the changes of MSC number, NC number, cell viability and potential contamination after bone marrow being processed in SECCS. Each experiment was repeated 3 times. Patients were followed up in outpatient where the bone healing was assessed.

**Table 1:** Baseline diseases of patients in the study.

Diseases	Number and percentage of patients
Nonunion	20 (47.6%)
Fresh fracture^ψ^	11 (26.2%)
Others	
Bone defect after hardware removal	2 (4.8)
Ankle arthrodesis revision	3 (7.1%)
Benign bone tumor	3 (7.1%)
Delayed union	2 (4.8)
Ankle deformity	1 (2.4%)

^ψ^A fresh fracture was defined as a fracture that occurred no earlier than 2 weeks before the surgical treatment.

### Bone marrow harvest

Bone marrow was harvested under electrocardiogram monitoring and anesthesia. About 80 mL bone marrow was harvested from the unilateral or bilateral anterior superior iliac spine in each patient. The harvesting strategy was the same as that reported in an earlier study^9^. To avoid mechanical damage to cells, a 12-gauge bevel needle was used. To prevent coagulation, 20-mL syringes were rinsed with heparinized saline (50,000 U heparin in 250 mL saline). During harvest, the time that the bone marrow was retained in the syringe was limited to 10 s. Then, bone marrow was temporarily stored in a sterile bag containing 6,000–7,000 U heparin. Bone marrow harvests were performed by an experienced doctor. Harvest volume and time were recorded.

After bone marrow harvest, the MSC/scaffold composites were prepared, and conventional surgery began simultaneously in the same operation room.

### Porous biomaterial

The biomaterial used in this study was degradable β-TCP (Bio-Lu Bioceramics, Shanghai, China). It comprises white granules having a diameter of 3–5 mm, porosity of 75% ± 10%, and mean pore size of 500 ± 200 μm. Adjacent pores are fully interconnected, and the mean size of the interconnected pores is 150 ± 50 μm. For each bone marrow sample, 5 g (13–15 mL) of β-TCP granules was put in the inner box of SECCS, serving as filtration material to screen and enrich BMSCs.

### SECCS

**Figure 7:**
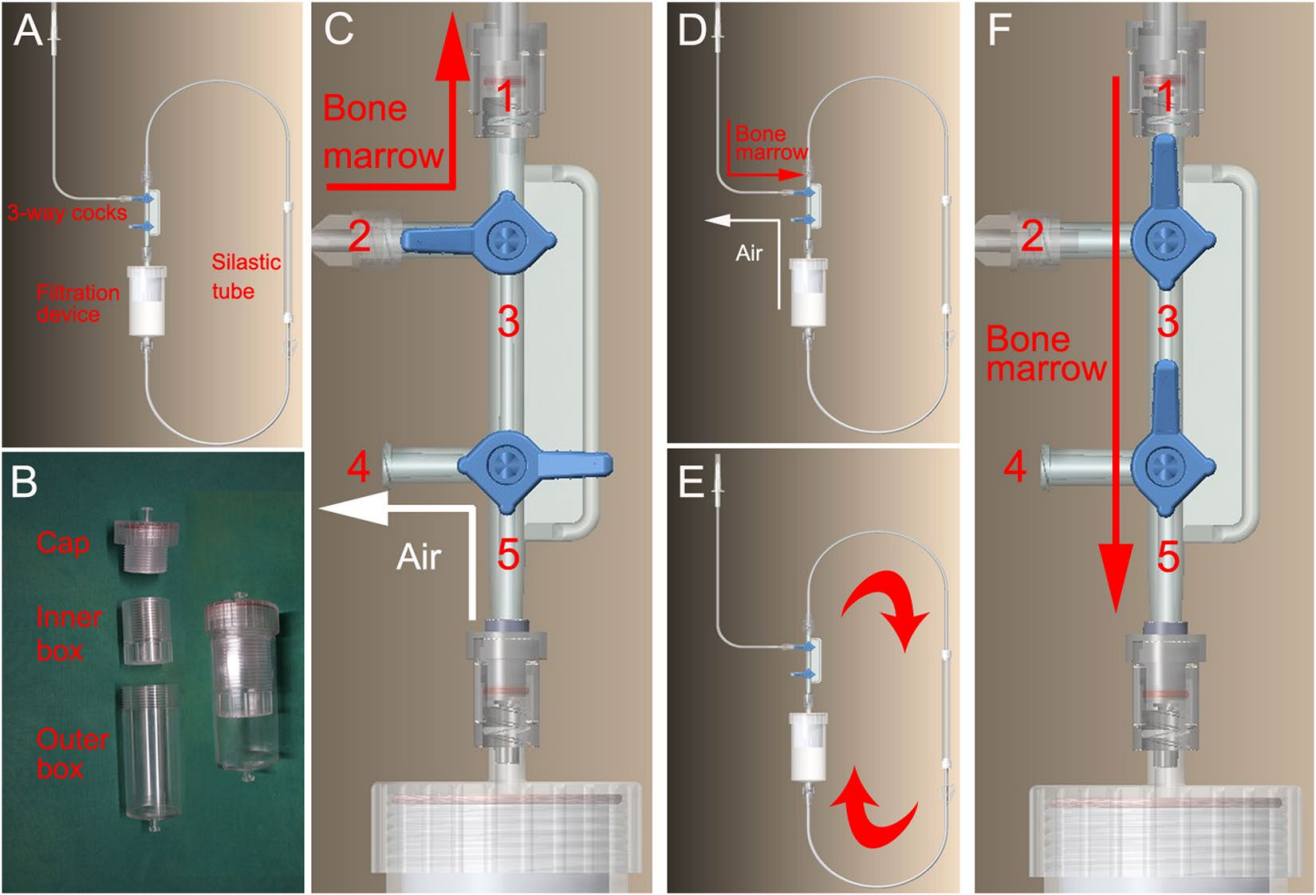
Structure and mechanism of the screen–enrich–combine(-biomaterials) circulating system (SECCS). (**A**) Plastic pipe: contains a detachable filtration device, a silastic tube, and 2 3-way cocks. (**B**) Detachable filtration device: consists of a cap, an inner box, and an outer box. (**C**,**D**) Bone marrow injection: by adjusting the 3-way cocks, *nozzle*.1 opens into *nozzle*.2, and *nozzle*.4 opens into *nozzle*.5, which means that *channel*.3 is closed. Bone marrow was poured into the pipe from *nozzle*.2, and air was exhausted via *nozzle*.4. (**E**,**F**) Formation of a sealed circulatory pipe: by adjusting the 3-way cocks, *nozzle*.1 is connected to *nozzle*.5 through *channel*.3. Thus, a sealed pipe is assembled for bone marrow circulation.

The SECCS consists of a peristaltic pump and a pipe system. The latter is a sealed plastic pipe, which contains a detachable filtration device, a 15-cm-long silastic tube, and 2 3-way cocks (Fig. 7A).

The functions of each part are as follows:

1. Peristaltic pump: exerts a force on the silastic tube to generate motive power for the circulation of bone marrow.
2. Filtration device: consists of a cap, an outer box, and an inner box (Fig. 7B). The inner box is designed for the placement of biomaterials. The outer box isolates the inner box and biomaterials from the external environment. The two boxes are linked to the cap by threads. Adequate threads between the cap and inner box can adjust the volume of the inner box according to the biomaterial volume required in the clinic (range, 9–85 mL).
3. Three-way cock: adjusts the flow direction of bone marrow for its injection and circulation.

The manipulation process was as follows:

1. Assembly: the pipe is placed on an aseptic preparation table. The filtration device is disassembled, porous biomaterials are put into the inner box, and the device is assembled together with the pipe on a sterile operation table (Fig. 7A).
2. Bone marrow injection: by adjusting the 3-way cocks (Fig. 7C), *nozzle*.1 opens into *nozzle*.2, and *nozzle*.4 opens into *nozzle*.5, which means *channel*.3 is closed. When the SECCS is in the perpendicular state, 60 mL bone marrow is poured into the pipe from *nozzle*.2, and air is ejected via *nozzle*.4 (Fig. 7C,D).
3. Formation of a sealed circulatory pipe: by adjusting the 3-way cocks (Fig. 7F), *nozzle*.1 is connected to *nozzle*.5 through *channel*.3. Thus, a sealed circulatory pipe is assembled (Fig. 7.E,F).
4. Filtration: the sealed pipe is placed on a supporting frame, and the silastic tube is embedded into the peristaltic pump. The peristaltic pump is switched on (70–80 Hz), and bone marrow is propelled in a circular manner in the circulatory pipe, to be filtered through the porous β-TCP particles. After 10–15 min, the MSC/β-TCP composites are prepared. Then, the inner box is taken off by a surgeon or an assistant, while maintaining sterile conditions. The MSC/β-TCP composite can be implanted into the graft site immediately.
5. Re-collection of bone marrow residue: the 3-way cocks are adjusted as described in step 2, and the residual bone marrow is aspirated from *nozzle*.2.

By the SECCS, the MSC/β-TCP composite is generated in the same operation room without interrupting the surgery. The inner wall of the pipe, inner box, and β-TCP are maintained in a sterile state during the cell processing.

### Quantitative estimation of MSCs

Before and after filtration in the SECCS, 10-mL samples of bone marrow were collected for culture for each sample. The samples were suspended in 2.5 mL alpha-minimum essential medium (Sigma, Santa Clara, California, USA) containing 10% fetal bovine serum (Hyclone, Logan, Utah, USA), 50 mg/mL sodium ascorbate (Sigma), 1% antibiotic/antimycotic, 10 mM glycerophosphate (Sigma), and 10^−8^ M dexamethasone (Sigma) and cultured in 6-well plates (0.2 mL per well, replicated in 3 wells) at 37 °C in a humidified atmosphere with 5% CO_2_. Medium was refreshed every 2 days. On day 10, alkaline phosphatase (ALP) staining was performed to stain the colony-forming units (CFUs) expressing ALP activity (ALP + CFU). The number of ALP + CFU indicates the number of MSCs in pre- or post-SECCS bone marrow^18,37^. ALP + CFU (diameter, ≥2 mm) numbers were counted by 2 independent experienced researchers. The ALP + CFU number was the mean of 3 wells. The difference between ALP + CFU numbers of pre- and post-SECCS bone marrow was determined for each patient. The formula to count the MSC screening-enrichment rate is (PRE _ALP+ CFU_ − POST _ALP+ CFU_)/PRE _ALP+ CFU_ × 100%.

### Nucleated cell count and cell viability

For each sample, 1 mL pre- and post-SECCS bone marrow was treated with red blood cell lysis buffer (BioTime, Shanghai, China). Then, the nucleated cells (NCs) were counted using a hemocytometer (Beckman Coulter, Brea, California, USA). Cell viability was assessed by the trypan blue exclusion rate (Vi-CELL XR Cell Viability Analyzer Software, Beckman Coulter). The difference between pre- and post-SECCS bone marrow was estimated for each patient.

### Bacteriological examinations

To monitor the aseptic conditions and safety of the SECCS, bacteriological examinations of pre- and post-SECCS bone marrow were performed.

### Confocal microscopy and ALP staining of MSC/β-TCP composites

In a 28-year-old male patient with nonunion, several MSC/β-TCP composites granules remained after surgery. A part of these granules was incubated in serum-free medium for 2 h and then washed with phosphate-buffered saline (PBS; Hyclone) to remove non-adhering cells. After fixation with paraformaldehyde (4%, pH 7.4), the granules were stained with 4ʹ,6-diamidino-2-phenylindole (Sigma) and phalloidin (Yeasen, Shanghai, China) for confocal microscopy observation. The remnant granules were cultured in 6-well plates. The culture condition is the same as mentioned the above, and MSC/β-TCP composite granules were imaged by confocal microscopy to observe cell adhesion and proliferation on the β-TCP on day 10.

### ALP staining of MSC/β-TCP composites

In another patient with bone cyst (a 43-year-old woman), some MSC/β-TCP composite granules remained after surgery. These granules were also subjected to osteogenic induction culture as previously mentioned. ALP staining was performed on day 10.

### Flow cytometry

In another patient with nonunion (a 25-year-old woman), some MSC/β-TCP composite granules remained after surgery. These granules were cultured in alpha-minimum essential medium containing 10% fetal bovine serum (FBS), 50 mg/mL sodium ascorbate and 1% antibiotic/antimycotic in standard condition for 3 weeks. Then, cells adhered on the β-TCP were digested using pancreatin (Sigma) and suspended in PBS containing 5% FBS. Cells were stained with anti-CD29-FITC, CD34-FITC, CD44-FITC, CD13-PE, and CD105-PE (Thermo Fisher Scientific, Waltham, MA, USA). These cells were analyzed using flow cytometer (Beckman Coulter CyAn ADP; Brea, CA, USA).

### Clinical documentation

Patients were surgically treated. Fresh fractures underwent open reduction and internal fixation. For nonunion patients, the unstable fractures were stabilized by fixation revision or augmentative fixation. All patients were grafted with MSC/β-TCP composites produced by SECCS. Changes in vital signs during bone marrow harvest were recorded. During hospital stay, wound complications (swelling, exudation, and suppuration) were observed. In addition, complications of bone marrow harvest sites were observed. Patients were discharged when vital signs were normal and surgical wounds were well controlled. Follow-up visits and radiographs were required regularly (in 1, 3, 6, 9, 12, and 24 months postoperatively). Two independent orthopedic surgeons evaluated the radiographs. The full bone healing was defined as that bone union could be observed on anterior-posterior and lateral radiography within 9 months.

### Statistical analysis

Differences of relative parameters (NC number, cell viability, and ALP + CFU number) between pre- and post-SECCS bone marrow were compared. Data normality was tested by the Kolmogorov–Smirnov test. For normally distributed data, the paired t-test was used, and for non-normally distributed data, Wilcoxon’s signed rank test was performed. Depending on data distribution, Student’s t-test or Wilcoxon’s signed rank test was performed to test differences in ALP + CFU for different sexes and diseases. Pearson’s correlation analysis was performed to test the correlation between ALP + CFU number and age. All the data were analyzed with SPSS 13.0 software (Statistical Package for the Social Sciences, Chicago, IL, USA) and are presented as the mean ± standard deviation (SD). A P value of less than 0.05 was considered statistically significant.

### Data availability statement

The authors declared that the data are available.
